# Profile and rural exposure for nursing and allied health students at two Australian Universities: A retrospective cohort study

**DOI:** 10.1111/ajr.12689

**Published:** 2021-02-10

**Authors:** Tony Smith, Keith Sutton, Alison Beauchamp, Julie Depczynski, Leanne Brown, Karin Fisher, Susan Waller, Luke Wakely, Darryl Maybery, Vincent L. Versace

**Affiliations:** ^1^ Department of Rural Health The University of Newcastle Taree NSW Australia; ^2^ Monash Rural Health Warragul Vic. Australia; ^3^ Department of Rural Health The University of Newcastle Moree NSW Australia; ^4^ Department of Rural Health The University of Newcastle Tamworth NSW Australia; ^5^ Monash Rural Health Latrobe Regional Hospital Traralgon Vic. Australia; ^6^ School of Medicine Deakin Rural Health Warrnambool Vic. Australia

**Keywords:** health professions, health workforce, Rural health, tertiary education

## Abstract

**Objective:**

Linking enrolment and professional placement data for students' from 2 universities, this study compares characteristics across universities and health disciplines. The study explores associations between students' location of origin and frequency, duration and type of placements.

**Design:**

Retrospective cohort data linkage.

**Setting:**

Two Australian universities, Monash University and the University of Newcastle.

**Participants:**

Students who completed medical radiation science, nursing, occupational therapy, pharmacy or physiotherapy at either university between 2 February 2017 and 28 February 2018.

**Interventions:**

Location of origin, university and discipline of enrolment.

**Main outcome measure(s):**

Main measures were whether graduates had multiple rural placements, number of rural placements and cumulative rural placement days. Location of origin, discipline and university of enrolment were the main explanatory variables. Secondary dependent variables were age, sex, socio‐economic indices for location of origin, and available placements.

**Results:**

A total of 1,315 students were included, of which 22.1% were of rural origin. The odds of rural origin students undertaking a rural placement was more than 4.5 times greater than for urban origin students. A higher proportion of rural origin students had multiple rural placement (56.0% vs 14.9%), with a higher mean number of rural placement days. Public hospitals were the most common placement type, with fewer in primary care, mental health or aged care.

**Conclusions:**

There is a positive association between rural origin and rural placements in nursing and allied health. To help strengthen recruitment and retention of graduates this association could be further exploited, while being inclusive of non‐rural students

**What is already known on this subject:**

- There is considerable government investment in building future rural health workforce capacity
- Extant literature focuses mostly on the link between medical education and pathways to rural practice and less so on nursing and allied health
- Nursing and allied health constitute a larger proportion of the health workforce than medicine, and more investigation of the non‐medical workforce is warranted

**What this study adds:**

- The study contributes to the body of knowledge about nursing and allied health professional education, emphasising the association between rural origin and rural placement experience
- In allied health and nursing, there is a strong association between growing up in a rural location and undertaking undergraduate rural professional placements
- The data linkage methodology making use of existing datasets has potential to be applied across other universities and disciplines

## 1 INTRODUCTION

Extensive literature has called attention to the inequitable distribution of the health workforce across Australia. Most research and reports have concentrated on the medical workforce, even though medicine is a relatively small proportion of the entire national registered health care workforce. Combined, nursing and midwifery make up 57% of nationally registered health professionals and dental and allied health professions (including pharmacy) constitute a further 26%, the remaining 17% being generalist and specialist medical practitioners.^1^ ^,p.282^ It is thus important to address the apparent gap in the literature by developing a greater understanding of factors that influence nursing and allied health practitioners to take up positions in rural, regional and remote locations.

A focus on rural placement opportunities for all health professional students in Australia has been a key strategy for building a sustainable and high‐quality multidisciplinary rural health workforce.^2^ University students enrolled in health professional degrees, many of who are based at metropolitan universities, undertake rural professional placements with varying levels of support through their ‘parent’ university. Rural and remote placements are also supported through the network of rural‐based University Departments of Rural Health (UDRHs) and Rural Clinical Schools, which are funded under Australian Government's Rural Health Multidisciplinary Training (RHMT) Program.^3^

Professional placement experiences provide opportunities for students to put theory into practice in a range of contexts and can be influential on students' decision‐making about their future career.^4^ During rural placements, students are able to develop an understanding of the nuances of rural practice in their discipline, as well as of the broader health care needs of rural communities^4^ and cultural protocols.^5^ There is potential for positive learning experiences on placement in a rural setting to stimulate students' interest in returning and practising in a rural location when they graduate.^6^, ^7^ New graduate rural practice work locations and rural graduate programs have been linked to high‐quality placement experiences.^4^, ^8^, ^9^, ^10^ Conversely, negative experiences can have detrimental effects on students, which can potentially deter them from rural practice.^11^, ^12^

As is the case with the majority of health workforce studies, most of the previous research exploring the nature of health professional students' placements and the association with their future practice intentions is limited to medicine, dating back several years,^11^, ^21^ although some studies have included nursing and allied health students.^7^, ^8^, ^9^, ^10^, ^28^ Existing evidence shows that rural background is apparently the most strongly influential factor on graduates' choice of rural practice location.^10^, ^11^, ^12^, ^21^, ^22^, ^23^ However, rural practice intentions at commencement of studies^14^, ^15^, ^16^, ^20^, ^21^ and exposure to rural health care settings during their education and training^8^, ^10^, ^14^, ^23^, ^24^, ^29^ are also key indicators of postgraduate rural practice. Indeed, some studies have suggested that rural placement experience might be a stronger predictor of rural practice intentions than rural background.^19^, ^24^ In one large‐scale Australia‐wide survey of allied health, nursing and medical students engaged in the RHMT Program, students who reported high levels of satisfaction with their rural placement experiences had 2.3 times higher odds of having a future rural practice intention.^10^

While the previous studies cited above have identified factors apparently linked to future rural practice intention, many were mono‐disciplinary, involved only students at a single university or geographical location, included only a small sample size or had other methodical limitations, posing issues of generalisability. However, all Australian universities routinely collect large amounts of data from and about their students, creating the opportunity for linkage of datasets and interrogation for a range of key variables. Consequently, the UDRHs associated with Monash University and the University of Newcastle have aggregated administrative datasets from both universities that include enrolment and clinical placement data for entire cohorts of nursing and allied health students. This increases the sample size and allows comparisons to be made between universities and across disciplines for key independent variables, including location of origin (rural vs urban) and a range of dependent variables. Therefore, the aim of the study reported herein was to combine data for graduate cohorts of nursing and allied health students from the 2 universities to:

- Compare profile characteristics of students between the universities and across disciplines
- Explore the associations between the location where students grew up or attended school and the extent and types of rural placement exposure during their degree.

## 2 METHODS

This was a retrospective cohort study using administrative student data from 2 Australian universities. Participants were students who completed the requirements of a nursing or allied health degree at the University of Newcastle or Monash University that would make them eligible for registration with the Australian Health Practitioners Regulation Agency (Ahpra).

### 2.1 Sample characteristics

The health professions included were medical radiation sciences (including diagnostic radiography, radiation therapy and nuclear medicine science), nursing, occupational therapy, pharmacy and physiotherapy. The sample included only students undertaking a single bachelor degree who were enrolled and completed their studies between 2 February 2017 and 28 February 2018, thus having graduated in 2018, regardless of the year that they commenced. Students with ‘international’ enrolment status (ie non‐domestic students) were excluded as rural background was defined using an Australian classification.^30^ Further exclusions were necessary to allow comparison between universities; thus, students enrolled in undergraduate degrees offered at only one of the universities were excluded, specifically podiatry and midwifery students from the University of Newcastle and paramedicine and psychology students from Monash University. The category of ‘nursing’ excluded midwifery, which is offered as a separate, single, undergraduate degree at the University of Newcastle but as a combined, double degree at Monash University.

### 2.2 Data collection

Routinely collected data were extracted from administrative datasets at each university. These data comprised student age at enrolment, sex, home address, secondary school location, and degree commencement and completion dates. In addition, data were also extracted for students' professional placement history over the duration of their studies. This data included placement location for each of their placements, dates and duration of placements, and the type of facility in which they were placed. Placement facilities were classified as follows: ambulance service; community health centre; community pharmacy; corrections facility; non‐government organisation; overseas; private hospital; private practice; public hospital; residential aged care; mental health service (even if that service was part of another, larger health facility, such as a public hospital). Placement facility types were of interest for descriptive purposes only.

### 2.3 Data analysis

Initially, researchers at each university cleaned, de‐identified and performed summary descriptive analysis on their own datasets. Subsequently, final analysis was undertaken on a combined dataset of linked records of students from both universities. Data were analysed using Software for Statistics and Data Science (Stata, version 15, StataCorp LLC) and Statistical Analysis Software (SAS, version 14.1, SAS Institute Inc).

The primary outcomes of interest were whether graduates had undertaken at least one rural placement during their degree, the number of rural placements undertaken and the cumulative number of days spent on rural placement. The main explanatory variable of interest was the students' location of origin, being where they grew up or, if different, where they went to school. Other covariates of interest were age, sex, socio‐economic index for their location of origin, number of placements available according to curriculum requirements, discipline or the university in which they were enrolled.

Descriptive data for student background, placement facility type and duration were analysed using medians or proportions. The variable for students ‘Location of origin’ was categorised using the Australian Statistical Geography Standard—Remoteness Area (ASGS‐RA) 2016 classification, which classifies the Australian land area into 5 categories.^30^ Students whose home address at enrolment was in a major city (RA1) were defined as being of ‘Non‐rural’ (or urban) origin, while those whose address was in inner regional, outer regional, remote or very remote (RA2‐5) were classified as ‘Rural’ origin. Where home address was not available, the address of the high school they attended was used for this categorisation.

The variable ‘Age at enrolment’ was dichotomised into < 21 years and ≥ to 21 years of age, the latter being considered ‘mature age’ entrants into Australian universities. ‘Location of origin’ was used to derive a secondary variable representative of ‘Socio‐economic disadvantage’, based on the students' home (or school) address being within a Statistical Area Level 2 (SA2)^31^ region in lowest 20% Socio‐Economic Indexes for Areas (SEIFA), which is based on the 2016 Index of Relative Socioeconomic Disadvantage (IRSD).^32^

Placement locations were also categorised using the ASGS‐RA classification^30^ as to whether they were ‘Non‐rural’ (or urban) or ‘Rural’, based on the primary address of the health service provider or placement facility. The variable ‘Number of placements’ was defined as the number of separate occasions a student spent a period of time at the facility for the purpose of work integrated learning according to curriculum requirements. ‘Number of placement days’ was calculated from the beginning and end date for all placements and summed over the duration of the students' enrolment. Differences between explanatory variables and placement outcomes by discipline and university were generated and assessed using chi‐squared or Kruskal‐Wallis tests of association.

Binary logistic regression was used to determine the association between ‘Rural origin’ and whether ‘At least one rural placement’ was undertaken during a student's degree. These data generated odds ratios (OR) with 95% confidence interval (CI). Generalised linear regression for a zero‐inflated negative binomial (ZINB) distribution was used to analyse the effects of ‘Rural origin’ on ‘Cumulative days of rural placement’. These data generated rate ratios (RR) with 95% CI. The ZINB method was used for the latter as there were potentially 2 processes at play; that is, whether a student went on a rural placement at all (0 days vs > 0 days) and the cumulative duration of rural placement exposure for students who had one or more rural placement. In addition, the outcome variable (days) was count data that had a high proportion of ‘zero values’ and the variance in the data was high compared to the mean, suggesting over‐dispersion.^33^ Goodness‐of‐fit tests (deviance, Pearson's chi‐squared and Akaike information criterion) also indicated that the ZINB model was a better fit for both nursing and allied health data than models using alternative distributions (negative binomial, Poisson, zero‐inflated Poisson).

Because the sample size in some disciplines, such as occupational therapy and pharmacy, was too small for regression analysis when broken down, by for example ‘Location of origin’, allied health students were combined into one group and analysed separately from nursing, with data from both universities combined. Explanatory variables with a *P*‐value < .25 in univariate models were entered into the base multivariate models. Determination of variables for inclusion in each arm of the ZINB model was assessed separately. All possible interactions were included, assessed successively and removed using a manual backward stepwise elimination technique,^34^ where *P* < .01, followed by assessment of the main effects, which were retained if assessed as a confounder or if *P* < .05. The model of best fit in final regression models was determined using Akaike information criterion (AIC), Hosmer and Lemeshow, Somers *D* and *c* tests of association of predicted probabilities and observed responses.^35^, ^36^ Regression analyses are presented for students of Rural vs non‐Rural origin, adjusting for other explanatory variables.

### 2.4 Ethics considerations

Ethics approval was obtained from both universities’ human research ethics committees (Monash University Human Research Ethics Committee: 7962, 29 August 2017; and the University of Newcastle Human Research Ethics Committee: H‐2017‐0332, 20 November 2017). Data were extracted by university administrative staff. Enrolment, graduation and placement data were linked using the students' enrolment identification (ID) numbers. Individual‐level student consent was waived as data collection fell within the privacy terms and conditions agreed to by all students on enrolment at each university.^37^, ^38^ Datasets containing student ID numbers were securely stored at each university separately and only de‐identified data were shared between universities.

## 3 RESULTS

A total number of 1776 students were potentially eligible to be included in the study, of which 156 (8.8%) were excluded because they were non‐domestic students. A further 305 potentially eligible students were excluded because the degrees they were enrolled in were not offered at both universities, as explained above in the description of the sample. As in Table 1, there were 1315 students that met the inclusion criteria, of which 829 (63.0%) attended the University of Newcastle and the remainder were enrolled at Monash University. Demographic variables considered to be potential factors affecting the location of student placements are shown in the table for students from both universities, combined and separately, for nursing, allied health overall and each separate allied health discipline. The median number of placements undertaken by students and median number of days of placement over the duration of their studies are also shown. Both varied between disciplines and universities and ‘Median placement days’ was taken as a proxy estimate of total available days of student placement according to curriculum requirements.

**TABLE 1:** Student background demographics, placement frequency and days by discipline for both universities combined and separately

Variable, n (%)	All Students	Nursing	All Allied Health^a^	Occupational Therapy	Pharmacy	Physiotherapy	Medical Radiation
Combined Universities							
Students (% of all students)	1315 (100%)	713 (54.2%)	602 (45.8%)	130 (9.9%)	65 (4.9%)	165 (12.5%)	242 (18.4%)
Female	1074 (81.7%)	648 (90.9%)	426 (70.8%)*	106 (81.5%)	46 (70.8%)	106 (64.2%)	168 (69.4%)
Age <21 y at enrolment	745 (56.7%)	306 (42.9%)	439 (72.9%)*	92 (70.8%)	29 (44.6%)	133 (80.6%)	136 (56.2%)
Socio‐economic disadvantage^b^	204 (15.5%)	112 (15.7%)	92 (15.3%)	10 (7.7%)	14 (21.5%)	20 (12.1%)	48 (19.8%)
Rural origin^c^	290 (22.1%)	141 (19.8%)	149 (24.8%)	31 (23.8%)	15 (23.1%)	39 (23.6%)	64 (26.4%)
Median placements* (IQR)	—	7 (6‐7)**	—	4 (4‐4)**	6 (4‐6)**	7 (7‐8)**	5 (5‐5)**
Median placement days* (IQR)	—	95 (90‐100) **	—	130 (92‐135)**	61 (60‐61)**	160 (160‐180)**	125 (110‐125)**
The University of Newcastle (UON)							
Students (% of UON students)	829 (100%)	452 (54.5%)	377 (45.5%)	82 (9.9%)	34 (4.1%)	78 (9.4%)	183 (22.1%)
Female	663 (80.0%)	404 (89.4%)	259 (68.7%)	63 (76.8%)	25 (73.5%)	46 (59.0%)	125 (68.3%)
Age <21 y at enrolment	434 (52.4%)	160 (35.4%)	274 (72.7%)	58 (70.7%)	26 (76.5%)	59 (75.6%)	131 (71.6%)
Socio‐economic disadvantage^b^	147 (17.7%)	83 (18.4%)	64 (17.0%)	8 (9.8%)	8 (23.5%)	15 (19.2%)	33 (18.0%)
Rural origin^c^	236 (28.5%)	115 (25.4%)	121 (32.1%)	26 (31.7%)	11 (32.4%)	29 (37.2%)	55 (30.1%)
Median placements* (IQR)	—	7 (7‐7)	—	4 (4‐4)	6 (6‐7)	8 (8‐8)	5 (5‐5)
Median placement days* (IQR)	—	95 (90‐97)	—	135 (135‐135)	61 (61‐61)	175 (175‐185)	110 (110‐115)
Monash University (MU)							
Students (% of MU students)	486 (100.0%)	261 (53.7%)	225 (46.3%)	48 (9.9%)	31 (6.4%)	87 (17.9%)	59 (12.1%)
Female	411 (84.6%)	244 (93.5%)	167 (74.2%)*	43 (89.6%)	21 (67.7%)	60 (69.0%)	43 (72.9%)
Age <21 y at enrolment	311 (64.0%)	146 (55.9%)	165 (73.3%)*	34 (70.8%)	3 (9.7%)	74 (85.1%)	5 (8.5%)
Socio‐economic disadvantage^b^	57 (11.7%)	29 (11.1%)	28 (12.4%)*	2 (4.2%)	6 (19.4%)	5 (5.7%)	15 (25.4%)
Rural origin^c^	54 (11.1%)	26 (10.0%)	28 (12.4%)	5 (10.4%)	4 (12.9%)	10 (11.5%)	9 (15.3%)
Median placements* (IQR)	—	7 (6‐7)	—	4 (4‐4)	4 (4‐4)	7 (7‐7)	6 (6‐6)
Median placement days* (IQR)	—	100 (90‐100)	—	92 (92‐92)	60 (60‐60)	160 (160‐160)	125 (125‐125)

Abbreviation: IQR, interquartile range.

^a^ Allied health includes physiotherapy, occupational therapy, medical radiation science and pharmacy.

^b^ Geographical location of origin in Statistical Area Level 2 in lowest 20% of Socio‐Economic Indexes for Areas (SEIFA; unknown: Allied health n = 2; Nursing n = 5).

^c^ Geographical location of origin in Australian Standard Geographical Classification—Remoteness Area (ASGS‐RA) regions 2‐5. (unknown: Allied health n = 2).

* Significant between allied health disciplines (*P* < .05, chi‐squared or Kruskal‐Wallis).

** Significant for disciplines between universities (*P* < .01, Kruskal‐Wallis).

Just in excess of four‐fifths (81.7%) of all the nursing and allied health students were female and a little more than a half (54.2%) were enrolled in a nursing degree at either university. Overall, the proportion of students of rural origin was 22%, with the University of Newcastle having significantly higher representation of rural origin students (Table 1). The majority of rural students were from inner regional areas (83.9%), with the remainder from outer regional locations. The number and duration of student placements varied significantly between disciplines and between universities for the same disciplines. Both overall and at each university, the median number of placement days was highest in physiotherapy and lowest for pharmacy. While nursing and pharmacy had a similar number of placements to physiotherapy, both had considerably fewer placement days and so the average duration of each placement was lower.

For nursing, there was a difference between the cohorts from the 2 universities in age at enrolment, with Monash University having a larger proportion of students < 21 years old (55.9% vs 35.4%; Table 1). For hometown socio‐economic disadvantage and rural origin, the University of Newcastle had the higher proportional representations for both of those variables (18.4% vs 11.1% and 25.4% vs 10.0%, respectively). Although the University of Newcastle had a higher proportion of male nursing students, there was no evidence of a difference between universities for sex. There were also no differences in the number or duration of nursing placements between universities.

In allied health, the University of Newcastle had a higher representation of students of rural origin than Monash University (32.1% vs 12.4%; Table 1). Pharmacy had the highest overall proportion of mature age students (55.4%), especially at Monash University where only about 10% of pharmacy students were <21 years of age. There were no other differences in demographic variables between the 2 universities for the allied health disciplines. Occupational therapy was the discipline with the highest proportion of female students at both universities, as well as for the 2 universities combined.

Table 2 summarises both the rural and Non‐rural types of placement sites or facilities that nursing and allied health students attended over the duration of their studies. The proportion of rural nursing placements was almost 10% lower than for allied health and for both by far the greatest proportion of both rural and urban placements were in public hospitals. Only 5.6% of allied health placements and 7.2% of nursing placements were in private hospitals, 92.7% of which were in urban locations. Some differences in placement type between nursing and allied health placements are clearly discipline‐related, such as for community pharmacy. No nursing placements were recorded in non‐government organisations, private practices or in schools. For nursing, there were proportionally more mental health and residential aged‐care placements in rural compared to non‐rural locations. For the allied health disciplines, few placements occurred in residential aged‐care facilities, with none in rural locations.

**TABLE 2:** Type and proportions of nursing and allied health placements by Rural vs Non‐rural location for both universities combined

Placement type	Nursing		Allied health^a^	
	Rural (%)	Non‐rural (%)	Rural (%)	Non‐rural (%)
Ambulance service	0 (0)	0 (0)	0 (0)	13 (0.5)
Community health centre	4 (0.6)	30 (0.7)	23 (2.9)	71 (2.9)
Community pharmacy	0 (0)	2 (0)	63 (7.8)	151 (6.2)
Mental health service	109 (15.2)	400 (9.7)	11 (1.4)	46 (1.9)
Non‐government organisation	0 (0)	0 (0)	9 (1.1)	30 (1.2)
Overseas	0 (0)	0 (0)	0 (0)	15 (0.6)
Private hospital	18 (2.5)	330 (8.0)	21 (2.6)	162 (6.7)
Private practice	0 (0)	9 (0.2)	153 (19.1)	447 (18.4)
Public hospital	497 (69.3)	2959 (71.7)	485 (60.4)	1332 (54.8)
Residential aged care	89 (12.4)	360 (8.7)	0 (0)	30 (1.2
School‐based service	0 (0)	0 (0)	26 (3.2)	126 (5.2)
Other	0 (0)	18 (0.4)	12 (1.5)	7 (0.3)
Total placements^b^	717 (14.8)	4128 (85.2)	803 (24.5)	2430 (74.2)

^a^ Allied health includes physiotherapy, occupation therapy, medical radiation science and pharmacy.

^b^ Percentages reflect Rural vs Non‐rural placements for nursing and allied health separately.

Table 3 shows the number and duration of rural placements undertaken by rural and non‐rural origin nursing and allied health students separately. Numbers and percentages are also given in each row category for all nursing and allied health students, as well as for all students combined. There was no difference in the proportion of rural vs non‐rural origin students that had a single rural placement; however, a higher proportion of students of rural origin compared to those of non‐rural origin had undertaken multiple (2 or more) rural placements in both nursing (56.0% vs 14.9%) and allied health (61.1% vs 28.2%). Further, the proportion of non‐rural origin students that had no rural placements was higher than for rural origin students in both nursing (63.9% vs 24.8%) and allied health (29.9% vs 8.1%). An association between rural origin and an increasing number of rural placements was apparent for both nursing and allied health students (*P* < .001; Mantel‐Haenszel chi‐squared test for trend).

**TABLE 3:** Number and duration of rural placements undertaken by individual students from both universities combined relative to students' geographical location of origin for nursing and allied health

Rural Placements	Students' Discipline and Location of Origin						All Students (%)
	Nursing (n = 711)^a^			Allied Health (n = 600)^a^, ^b^			
	Rural Origin (%)	Non‐Rural Origin (%)	All Nursing (%)	Rural Origin (%)	Non‐Rural Origin (%)	All Allied Health (%)	
Number of placements							
0*	35 (24.8)	364 (63.9)	399 (56.1)	12 (8.1)	135 (29.9)	147 (24.5)	546 (41.6)
1	27 (19.2)	121 (21.2)	148 (20.8)	46 (30.9)	189 (41.9)	235 (39.2)	395 (29.2)
2 or more*	79 (56.0)	85 (14.9)	164 (23.1)	91 (61.1)	127 (28.2)	218 (36.3)	382 (29.1)
Total students	141 (100.0)	570 (100.0)	711 (100.0)	149 (100.0)	451 (100.0)	600 (100.0)	1311 (100.0)
Duration of placements							
0 wk	35 (24.8)	364 (63.9)	399 (56.1)	12 (8.1)	135 (29.9)	147 (24.5)	546 (41.6)
>0 ≤2 wk	14 (9.9)	68 (11.9)	82 (11.5)	4 (2.7)	48 (10.6)	52 (8.7)	134 (10.2)
>2 ≤4 wk	13 (9.2)	49 (8.6)	62 (8.7)	9 (6.0)	43 (9.5)	52 (8.7)	114 (8.7)
>4 ≤8 wk	25 (17.7)	51 (9.0)	76 (10.7)	46 (30.9)	116 (25.7)	162 (24.0)	238 (18.2)
>8 wk*	54 (38.3)	38 (6.7)	92 (12.9)	78 (52.4)	109 (24.2)	187 (31.2)	279 (21.3)
Total students	141 (100.0)	570 (100.0)	711 (100.0)	149 (100.0)	451 (100.0)	600 (100.0)	1311 (100)

^a^ Excludes those where either placement location or geographical origin were not known (n = 4).

^b^ Allied health includes physiotherapy, occupation therapy, medical radiation science and pharmacy.

* In both nursing and allied health *P* < .001 (Mantel‐Haenszel chi‐squared test for trend).

Similarly, compared to non‐rural origin students, higher proportions of students of rural origin had 8 or more weeks of rural placement over the duration of their studies in both nursing (38.3% vs 6.7%) and allied health (52.4% vs 24.2%). The association between rural origin and longer duration of rural placements was also apparent for both groups (*P* < .001; Mantel‐Haenszel chi‐squared test for trend). Meanwhile, the proportion of nursing students who had rural placements of 2 weeks or less was 26.3% compared to 11.5% of allied health students.

### 3.1 Regression analyses

The summary results of regression analyses for ‘At least one rural placement’ and ‘Cumulative days of rural placement’ are given for nursing in Table 4 and allied health in Table 5. Results for the rural origin cohort are inclusive of both inner and outer regional students. For readers who desire more detail, full results of the final regression models for inner and outer regional cohorts are available on request to the corresponding author.

**TABLE 4:** Multivariate regression for ‘At least one rural placement’ and ‘Cumulative rural placement days’ for nursing students of both Universities, 2014‐2017

Variables Remaining in Final Models	Univariate Model			Multivariate Model		
Model 1. Binary Logistic Regression ‘At least one rural placement’ (n = 711)	Odds ratio	Lower 95% CI	Upper 95% CI	Odds ratio	Lower 95% CI	Upper 95% CI
Location of home address at enrolment						
Rural vs major cities^a^, ^b^	4.88*	2.62	9.10	4.64*	2.86	7.52
University of attendance						
University of Newcastle vs Monash University^a^	14.29*	9.24	22.10	13.38*	8.54	21.00

Model 2. Generalised Linear Regression^c^ ‘Cumulative days of rural placement’ (n = 711)	Rate ratio	Lower 95% CI	Upper 95% CI	Rate ratio	Lower 95% CI	Upper 95% CI
Location of home address at enrolment						
Rural vs major cities^a^, ^b^	1.81*	1.56	2.10	1.75*	1.50	2.02
University of attendance						
University of Newcastle vs Monash University^a^	2.07*	1.57	2.73	1.85*	1.43	2.40
Age at enrolment						
‘21 y or more’ vs ‘<21 y’^a^	1.13	0.96	1.32	1.19*	1.02	1.39
SEIFA‐IRSD socio‐economic disadvantage of home address at enrolment^d^						
‘Most disadvantaged quintile’^e^ vs ‘all others'^a^	1.14	0.94	1.40	1.32	0.98	1.79

^a^ Reference group.

^b^ ASGS‐RA_2016 Classifications: 1 = Major cities; 2‐5 = Rural.

^c^ Zero‐inflated negative binomial regression. Results for negative binomial component of the model only. Location of ‘Home address at enrolment’ (*P* < .001) and ‘University of attendance’ (*P* < .001) remained significant in the final zero model.

^d^ Socio‐Economic Index for Areas—Index of Relative Socio‐Economic Disadvantage: Significant interaction between ‘SEIFA‐IRSD’ and ‘Age at enrolment’ (*P* = .005).

^e^ Students with a home address at enrolment in a statistical local area 2 (SA2) with a SEIFA‐IRSD of 1 or 2. (‘all others’ ranged from 3 to 10).

* *P* < .05.

**TABLE 5:** Multivariate regression for ‘At least one rural placement’ and ‘Cumulative days of rural placement’ for allied health students of both Universities, 2014‐2017

Variables Remaining in Final Models	Univariate Model			Multivariate Model^c^, ^d^		
Model 1. Binary logistic regression: ‘At least one rural placement’ (n = 600)	Odds ratio	Lower 95% CI	Upper 95% CI	Odds ratio	Lower 95% CI	Upper 95% CI
Location of home address at enrolment						
Rural vs major cities^a^, ^b^	4.88*	2.62	9.10	4.57*	2.36	8.87
Available placements over entire degree						
Increase for each extra available or required placements	1.54*	1.34	1.77	1.74*	1.30	2.31

Model 2. Generalised linear regression ‘Cumulative days of rural placement’ (n = 600)	Rate ratio	Lower 95% CI	Upper 95% CI	Rate ratio	Lower 95% CI	Upper 95% CI
Location of home address at enrolment						
Rural vs major cities^a^, ^b^	1.40*	1.23	1.58	1.25*	1.12	1.40
University of attendance						
University of Newcastle vs Monash University^a^	1.62*	1.44	1.83	1.71*	1.53	1.91
Placement experience weeks available over entire degree						
‘More than 120 d’ vs ‘120 d or less’^a^	1.60*	1.43	1.79	1.72*	1.55	1.91

^a^ Reference group.

^b^ ASGS‐RA 2016 Classifications: 1 = Major cities; 2‐5 = Rural.

^c^ Multivariate logistic regression model also adjusts for ‘Discipline’ (*P* < .001), ‘University’ (*P* = .183) and an interaction between ‘Discipline’ and ‘University’ (*P* = .003).

^d^ Zero‐inflated negative binomial regression. Results for negative binomial component of the model only. Location of ‘Home address at enrolment’ (*P* < .001), ‘Discipline’ (*P* < .001), ‘University of attendance’ (*P* = .010) and ‘Available placements over entire degree; (*P* = .001) remained significant in the final zero model.

* *P* < .05.

For nursing students, being of rural origin and being enrolled at the University of Newcastle were the only significant explanatory variables associated with whether a student had a rural placement or not (*P* < .001). Adjusting for university of enrolment, the odds of nursing students of rural origin having had at least one rural placement were more than four‐and‐a‐half times greater than for urban origin students (OR = 4.64; 95% CI = 2.86‐7.52; Table 4). Rural origin, attending the University of Newcastle, and older age at enrolment (≥21 years) were all associated with greater number of rural placement days. Socio‐economic disadvantage was an effect modifier due to its interaction with age. Adjusting for these variables, rural origin nursing students had 75% more rural placement days than urban origin nursing students (RR = 1.75; 95% CI = 1.51‐2.03; Table 4).

Table 5 shows regression analysis results for the allied health students. Rural origin, the available or required number of placements and the specific allied health discipline were associated with students having had a rural placement. The university of enrolment was an effect modifier that remained in the final model due to its interaction with ‘allied health discipline’. Adjusting for these variables, allied health students of rural origin had more than four‐and‐a‐half times higher odds of having had at least one rural placement compared to those of urban origin (OR = 4.57; 95% CI = 2.36‐8.87; Table 5). Rural origin, greater number of available placement days (>120 days) and being enrolled at the University of Newcastle were all associated with greater cumulative number of days of rural placement. The adjusted model shows that allied health students of rural origin had 25% more days on rural placement than their urban origin counterparts (RR = 1.25; 95% CI = 1.12‐1.40; Table 5).

**FIGURE 1:**
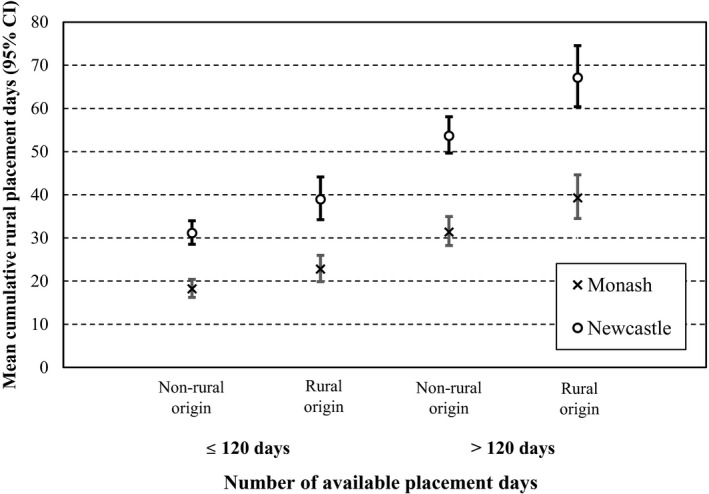
Mean rural placement days (95% CI) for allied health students of Rural or Non‐rural origin by available or mandated placement days within discipline and university of attendance

In Figure 1, the estimated mean number of days of Rural placement for allied health students is compared for each university and for Rural vs non‐Rural origin, as well as stratified by the available or required days of placement (≤120 days vs >120 days). The cut‐off of 120 days was based on examination of medians days of placement shown in Table 1 as well as knowledge about allied health curriculum requirements and represents an average of 6 weeks of placement per year across a four‐year degree. It is apparent that, on average, students from the University of Newcastle had a higher number of rural placement days than Monash students. Generally, students of rural origin from either university averaged a higher number of rural placement days than non‐rural origin students. The number of available days of placement time also correlates positively with mean estimates for rural placement days.

There was no evidence of interaction between the number of available or required placement days and rural origin for either university. Thus, despite students at the University of Newcastle completing more days of rural placement, the rate ratio between students of rural vs non‐rural origin was the same for both universities (RR = 1.25; 95% CI = 1.12‐1.40, as in Table 5), independent of the number of available placement days. Similarly, rate ratios for duration of rural placements by university or by available placement days were independent of other factors in the model. For those with more than 120 available placements days, the number of rural placement days was 1.72 times higher than for those with 120 or less days of available placement with no difference between rural and non‐rural students. Location of origin was not an effect modifier; however, as this was a zero‐inflated model, it only applies to those who had rural placements (a non‐zero outcome).

## 4 DISCUSSION

The key finding of this study is that rural origin students have greater odds of undertaking rural placements and having a greater number of cumulative rural placement days than those who come from major cities. Some student characteristics differed between the 2 universities in this study. Compared to Monash University students, a greater proportion of the University of Newcastle students were of rural origin and, particularly in nursing, tended to be older and from more socio‐economically disadvantaged areas. The university attended, however, was not a significant factor in students' rural placement experience. In general, a smaller proportion of nursing students had rural placements and, while placement type is to some extent professional‐specific, overall the predominant type was public hospital placements.

This study adds to the body of evidence that geographical location of origin makes a difference to decisions and choices that nursing and allied health students make about the locations for their professional placement experiences.^4^, ^11^ Such decisions and choices affect the degree of exposure they receive to rural practice, as well as their experience of the broader aspects of rural lifestyle, values and culture.^7^, ^8^, ^22^ The extent to which students can choose their preferred placement locations might be limited while they are studying^12^ due to university or discipline equity policies. Nevertheless, rural origin students have higher odds than urban origin students of undertaking rural placements, which might further increase their commitment to becoming rural practitioners in the future. In the context of the ongoing need to build rural health workforce capacity, the Australian government's policies and strategies,^2^, ^39^ including in the RHMT Program,^3^ include recruiting students of rural origin into university as a key equity initiative. Australian universities award bonus admission points in order to recruit students that meet certain criteria, targeting those of rural origin, low socio‐economic status or those with Aboriginal or Torres Strait Islander background.

The current evidence suggests that students of rural origin are most likely to enter into rural practice when they complete their studies.^10^, ^12^, ^13^, ^16^ However, in general, rural students come from relatively low socio‐economic backgrounds and might require ongoing support throughout their studies to ensure successful completion.^40^ Consequently, affirmative action should extend beyond merely recruiting rural origin students into health professional degrees at university. There is a need to target them throughout their studies, encouraging, prioritising and supporting their preferences for rural placement locations, with the aim of further strengthening their rural practice intentions. However, undertaking rural professional placements as part of their studies can carry a considerable financial, as well as social imposition.^41^ While all students should be encouraged to consider rural practice, given the general predisposition of rural origin students to rural career paths, it seems logical to nurture such aspirations by capitalising on rural placement and training opportunities.

In addition to the overall finding that students' rural origin is closely linked to rural placement location, some of the finer details can also be of importance. The study found clear differences between the 2 universities in location of origin of both nursing and allied health students. Compared to Monash University students, a higher proportion of the University of Newcastle students came from a rural background, which equated with being from a location classified as being socio‐economically disadvantaged.^40^ Monash University nursing students also tended to be younger at enrolment, with a higher proportion of University of Newcastle students falling into the mature age category. These differences might be due to the fact that, while Monash University is larger with campuses principally located in Melbourne, the University of Newcastle has a smaller metropolitan base and a catchment area that includes regional and rural locations.

The odds of rural origin nursing students undertaking rural placements was similar to allied health students, at just over 4.5 times that of non‐rural origin students. However, compared to allied health, rural origin nursing students who had at least one rural placement had a comparatively higher proportion of rural placement days compared to urban origin peers. This calls attention to the number of mandated placement days specified for each discipline under discipline‐specific accreditation criteria. It is noted that, compared to most allied health disciplines, with the exception of pharmacy, nursing had a relatively low number of total required placement days, so consequently the number of rural placement days would be expected to be low for most nursing students. This is supported by the data in Table 2, where the proportion of total rural placements in nursing was appreciably lower than for the combined allied disciplines. In addition, this relationship was well demonstrated within the allied health disciplines at both universities, where the number of rural placements days fell with the lower total number of available placement days. The case for specifying a mandatory number of rural placements or placement days, which is advocated by some^4^, ^42^ but not by others,^9^ should be the focus of future research. It is acknowledged that professional placement allocation is complex, however. Confounders might include a perception that metropolitan practice is somehow better and that meeting supervision requirements is challenging in rural settings, as is negotiating and maintaining agreements with rural placement providers.^43^

Overall, the types of rural placements were somewhat limited, with a strong bias towards the acute care public hospital system, which provided in excess of 60% of rural placements for both nursing and allied health students. This is not surprising, in that public hospitals offer a wide case‐mix and varied learning opportunities^10^; however, it seems contrary to the global shift in models of care and, therefore, the need to offer student learning experiences away from acute care towards more integrated, community‐based and primary care settings.^44^ Evidence from this study shows relatively little use of residential aged care, private hospitals, community health and mental health services. The imperative for the health care system in the future to better support the needs of those with chronic health conditions, including mental illness, especially in rural locations, suggests a need to diversify student placement opportunities. This is supported by the recent review of the nursing accreditation standards, which acknowledged the importance of education in mental health and care of older persons in particular.^45^ ^,p. 16^ While there might be some limitations, such as there being fewer private hospitals in rural areas,^46^ many rural communities would be well suited to integrated models of student placements that extend beyond the public hospitals and into rural community settings. A further barrier is that many such non‐acute potential placement settings in rural locations do not employ appropriately qualified practitioners to supervise students, although alternative interprofessional or remote models of supervision could be developed.

### 4.1 Strengths and limitation

This study points to the potential of linking reservoirs of data routinely collected by Australian universities to answer research questions and inform national policy directions, such as in relation to the maldistribution of the health workforce. Linking university administrative data sets has demonstrated that rural origin students are likely to have greater exposure to rural practice, potentially strengthening their resolve to become rural practitioners. Generalisability of the findings is limited because data included students from only 2 universities; yet, this study demonstrates the potential to link data sets across multiple universities for large‐scale data analysis.

Potentially eligible health professional students from each university were excluded if an equivalent degree was not offered at both universities. Other limitations include the small sample size of some allied health cohorts, preventing more detailed analysis. Exclusion of students undertaking graduate‐entry or double degrees also limited sample size, though differences in their curriculum requirements might have skewed the results if they were included. Furthermore, the study only included those professions regulated through Ahpra, thereby excluding disciplines integral to sustainable, high‐quality health care, such as speech pathology, social work and dietetics.

## 5 CONCLUSIONS

Coming from a rural area is an important predictor of rural practice and as part of the ongoing commitment to the development of future rural health workforce capacity, it is important to better understand and strengthen the pathways between rural origin, education and future health professional practice. Among the nursing and allied health students included in this study, there was a strong positive association between being of rural origin and the undertaking rural placements. There is an opportunity to build further on this through affirmative action. Recruiting health professional students into university from rural locations is important; however, ongoing rural exposure has potential to enhance their appreciation of rural work and encourage them to consider rural practice as a viable option after they graduate, rather than migrating to the cities.

The results of this study suggest some other opportunities for improvement. Innovation and development of new types of rural professional placement should be considered, particularly in the relatively underused primary and community health care and residential aged‐care sectors, where there is a future expectation of the need for substantial workforce growth and development. This study also demonstrated a potential to up‐scale student and graduate health professional research through cross‐jurisdictional, inter‐university collaborations and making use of routinely collected data.

## AUTHOR CONTRIBUTIONS

Tony Smith involved in conceptualisation; investigation; methodology; project administration; supervision; writing original draft; writing reviewing editing. Keith Sutton involved in conceptualisation; investigation; methodology; project administration; supervision; writing reviewing editing. Alison Beauchamp and Julie Depczynski involved in data curation; formal analysis; investigation; validation; visualisation; writing reviewing editing. Leanne Brown, Karin Fisher, Susan Waller, and Luke Wakely involved in validation and writing reviewing editing. Darryl Maybery, Vincent L. Versace, involved in project administration; validation; writing reviewing editing.
